# Isolation, Expression, and Characterization of a *Hydroperoxide Lyase* Gene from Cucumber

**DOI:** 10.3390/ijms141122082

**Published:** 2013-11-07

**Authors:** Xu-Hua Wan, Shu-Xia Chen, Cong-Ying Wang, Ran-Ran Zhang, Si-Qiong Cheng, Huan-Wen Meng, Xiao-Qing Shen

**Affiliations:** Key Laboratory of Horticultural Plant Germplasm Resources Utilization in Northwest China, College of Horticulture, Northwest A&F University, Yangling 712100, China; E-Mails: wanxh@nwsuaf.edu.cn (X.-H.W.); wangcongying0116@gmail.com (C.-Y.W.); zr19890318@nwsuaf.edu.cn (R.-R.Z.); chengsiqiong@gmail.com (S.-Q.C.); menghuanwen2005@gmail.com (H.-W.M.); shenxiaoqing@nwsuaf.edu.cn (X.-Q.S.)

**Keywords:** *Cucumis sativus* (cucumber), hydroperoxide lyase, gene cloning, sequence analysis, expression mode, C6 and C9 aldehyde content, hydroperoxide lyase activity

## Abstract

A full-length cDNA coding for hydroperoxide lyase (*CsHPL*) was isolated from cucumber fruits of No. 26 (Southern China type) and No.14-1 (Northern China type), which differed significantly in fruit flavor. The deduced amino acid sequences of *CsHPL* from both lines show the same and significant similarity to known plant HPLs and contain typical conserved domains of HPLs. The recombinant *CsHPL* was confirmed to have 9/13-HPL enzymatic activity. Gene expression levels of *CsHPL* were measured in different organs, especially in fruits of different development stages of both lines. The HPL activities of fruit were identified basing on the catalytic action of crude enzyme extracts incubating with 13-HPOD (13-hydroperoxy-(9*Z*,12*E*)-octadecadienoic acid) and 13-HPOD + 9-HPOD (9-hydroperoxy-(10*E*,12*Z*)-octadecadienoic acid), and volatile reaction products were analyzed by GC-MS (gas chromatography-mass spectrometry). *CsHPL* gene expression in No. 26 fruit occurred earlier than that of total HPL enzyme activity and 13-HPL enzyme activity, and that in No. 14-1 fruit was consistent with total HPL enzyme activity and 9-HPL enzyme activity. 13-HPL enzyme activities decreased significantly and the 9-HPL enzyme activities increased significantly with fruit ripening in both lines, which accounted for the higher content of C6 aldehydes at 0–6 day post-anthesis (dpa) and higher content of C9 aldehydes at 9–12 dpa.

## 1. Introduction

For a long period, researchers have paid considerable attention to improving the commercial characteristics of cucumbers, such as quality, yield, and disease resistance, whereas there are few reports on its nutritional quality and flavor, especially the mechanisms underlying the formation of volatile compounds and pathways that appeal to consumers’ senses [1]. Lipoxygenase (LOX) catalyzes the stercospecific oxygenation of position 13 or 9 of either linoleic acid or linolenic acid to produce linoleic acid hydroperoxides, such as 13(*S*)-hydroperoxy-(9*Z*,11*E*)-octadecadienoic acid (13-HPOD) and 9(*S*)-hydroperoxy-(10*E*,12*Z*)-octadecadienoic acid (9-HPOD), or linolenic acid hydroperoxides such as 13(*S*)-hydroper-oxy-(9*Z*,11*E*,15*Z*)-octadecatrienoic acid (13-HPOT) and 9(*S*)-hydroperoxy-(10*E*,12*Z*,15*Z*)-octadecatrienoic acid (9-HPOT). These compounds are cleaved by hydroperoxide lyase (HPL) to form aldehydes and relevant alcohols, resulting in the fragrance of cucumber fruit [2]. Recent studies suggested that this is the only metabolic pathway of aldehyde formation in cucumber [3]. HPL is one of the key enzymes in the LOX-HPL pathway that catalyzes 13-HPOD (13-HPOT) or 9-HPOD (9-HPOT) to produce *cis*-C6 or C9 aldehydes and oxyacids, and then converts them to *trans*-aldehydes, *trans*-oxyacids, and alcohol dehydrogenase (ADH) with the help of isomerase (IF) and alcohol dehydrogenase (ADH) to form alcohol and ester derivatives [4,5]. Aldehydes, alcohols, and esters are the sources of fragrance in leaves and fruits [3]. Therefore, understanding the sequence characteristics and expression mode of the *HPL* gene, as well as its relationship with specific enzyme activities, is important for gaining useful insights to determine the mechanism of aldehyde formation and improving the quality of cucumber fruit.

HPL belongs to the cytochrome P450 (CytP450) protein family. In plants, HPLs can be divided into 3 types according to the specificities of the substrate: 13-HPL, 9-HPL, and 9/13-HPL. 13-HPL specifically catalyses 13-HPOD or 13-HPOT to produce C6 and C12 compounds, respectively [*6*]; 9-HPL mainly catalyzes 9-hydroperoxides to form C9 aldehydes [7]; and 9/13-HPL can use both 9- and 13-hydroperoxides as substrates to produce C6 and C9 aldehydes [8]. The *13-HPL* genes that specifically catalyze the 13-site constitute the *CYP74B* sub-gene family, whereas the *9/13-HPL* genes that can catalyze both the 13-site and the 9-site constitute the *CYP74C* sub-gene family. Thus far, 3 *HPL* genes have been cloned from different plants, most of which harbor *13-HPL* genes, including alfalfa [9], pepper [10], tomato [11], potato [12], cucumber [8], and melon [13]. The *9/13-HPL* genes have also been cloned from cucurbitaceous vegetables, legumes (*Medicago truncatula*) and rice [14]. Three types of *HPL* genes—*13-HPL*, *9-HPL*, and *9/13-HPL*—have been reported in cucumber, in which 9/13-HPL acts on 9-HPOs and 13-HPOs. The HPL enzyme is closely related to the production of fragrance and pleasant “green notes” in cucumber [2,13] and the different ratios of C6 and C9 aldehydes result in the different flavors of cucumber: a higher content of C6 aldehyde provides a more “green grass” flavor, while a greater content of C9 aldehyde enhances the unique “cucumber-like” flavor [15].

The expression of *HPL* genes have been reported to be developmentally regulated and tissue specific. For instance, the expression of *13-HPL* in older tomato leaves is lower than that in younger leaves and is absent in stems and immature fruits [16]. On the other hand, expression levels in 9-day-old olives are lower than those in 28-day-old mature fruits and functional leaves [17]. Higher *HPL* expression has been found during the early developmental stages of fruits and floral organs such as grape berries [18,19] and in young potato leaves; its expression was rarely noted in old potato leaves [20]. The expression levels of 13-HPL in *Arabidopsis* leaves and inflorescence are lower and can be induced by damage [21]. In cucumber, the highest *9/13-HPL* expression level has been reported in 9-day-old seedling hypocotyls, followed by 9-day-old seedling cotyledons and 30-day-old female flowers, whereas its expression was considerably lower in the tendrils and leaves [22]. *HPL* is also known to participate in fruit maturation [17], seed germination [23], and defense responses [24–26]. It is also actively involved in the pathway of volatile formation in fruits and leaves [17,27]; in particular, the derivatives of the hydroperoxide lyase pathway such as aldehydes and dodecenedioic acids also showed a role as signals after wounding and simulated herbivory of plants [28].

Previous studies have focused on the involvement of the *HPL* gene in defense responses and stress, as well as in the production of aldehydes. *HPL* genes involved in aroma volatile biosynthesis have been studied and the relationship between *HPL* gene expression and enzymes responsible for fruit volatile formation has been reported in guava, melon, peach, tomato and almond [13,29–32]. However, the relationship among *HPL* gene expression, enzyme activities, and fruit aldehydes formation during fruit development is still unclear [3,8,10,30]. In this study, we reported molecular cloning of *HPL* gene of cucumber, and catalytic characteristics and expression in developing cucumber fruits. The capability of crude enzyme extracts of cucumber fruits C6 and C9 aldehyde compounds were measured using GC-MS (gas chromatography-mass spectrometry) analysis of crude enzyme extracts of cucumber fruits at different stages. Prokaryotic expression of *CsHPL* and C6 and C9 volatile compounds cleaved by recombinant protein were measured using GC-MS analysis from catalytic action and confirmed the 9/13-HPL enzyme activity characteristics of *CsHPL*. In addition, the relationship among gene expression mode, hydroperoxide lyase activity, and C6 and C9 contents in fruit at different development stages was analyzed. The obtained result would provide a basis for further study of the accumulation of C6 and C9 compounds in cucumber fruits.

## 2. Results and Discussions

### 2.1. Cloning and Sequences Analysis of the *CsHPL* Gene

**Figure 1:**
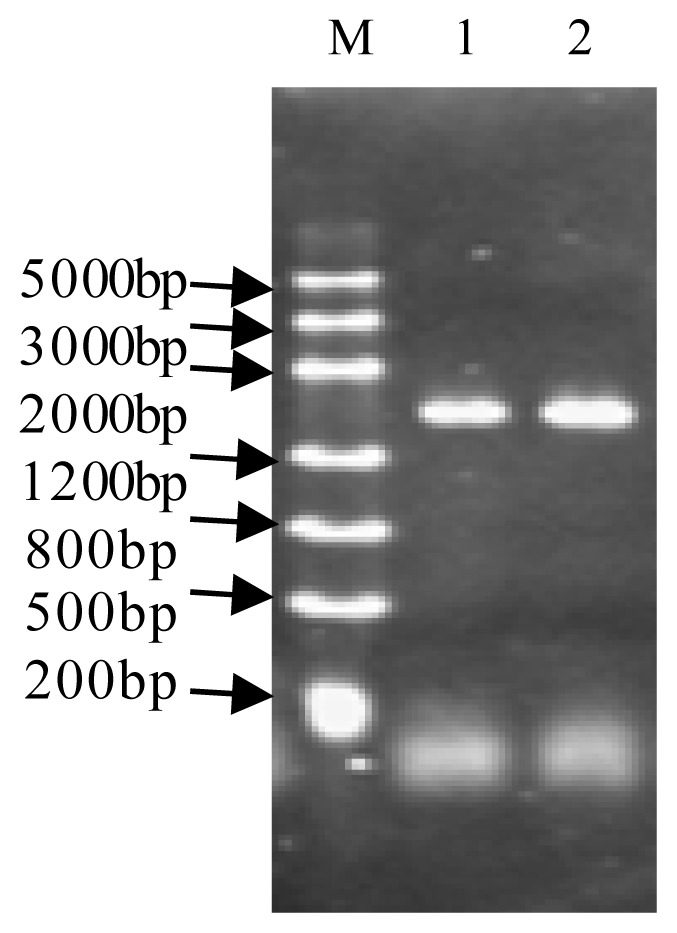
PCR product of *CsHPL* in cucumber. **M**: DL 5000 Marker; **1**: PCR product of No. 14-1; **2**: PCR product of No. 26.

A pair of specific primers, *HPL-*F and *HPL-*R, was designed according to the sequence of the *HPL* genes (Accession Number AF229811). The primer sequences were as follows: *HPL-*F, 5′-ATGGCTTCTTCCTCCCCTGAA-3′; *HPL-*R, 5′-TTAGCTTCTAAACCGAAGCGG-3′. After total RNA had been extracted from the young cucumber fruit of No. 26 and 14-1, the first strand of cDNA template was reversely transcribed and amplified by *HPL-*F and *HPL-*R primers, generating ~1400 bp fragments (Figure 1). The target fragments were recovered and purified, then attached to T-easy vectors. Three positive clones were selected for each inbred line and sent to Sangon Biotech Co., Ltd. (Shanghai, China) for sequencing.

**Figure 2:**
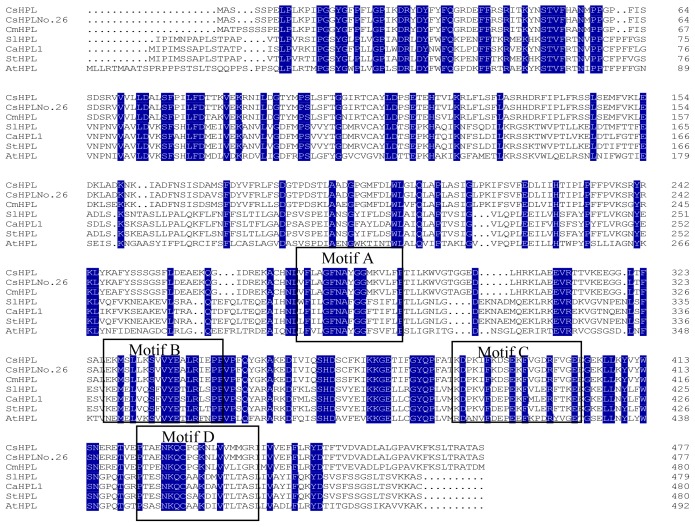
Amino acid sequence homology between cucumber and other HPL. *CsHPL* from No. 26; *CsHPL*: AAF64041.1, *Cucumis sativus; Cm9-HPL*: AAK54282.1, *Cucumis melo; SlHPL*: CAB43022.1, *Solanum lycopersicum; CaHPL1*: AAK27266.1, *Capsicum annuum; StHPL*: ACT64589.1, *Solanum tuberosum; Cl13-HPL*: AAU12570.1, *Citrullus lanatus; At13-HPL*: AAC69871.1, *Arabidopsis thaliana.*

According to the sequencing results, the fragment size was 1437 bp. The sequence of *CsHPL* in No. 26 was almost the same as that in No. 14-1 except for nucleotide “A” at position 1392 in No. 26 which is a “C” at 1392 position in No. 14-1; the deduced amino acid sequences of *CsHPL* from No. 26 and 14-1 were the same and shown to belong to the *CytP450* gene family according to the conserved protein regions, which has a calculated molecular mass of 53,926 Da and an isoelectric point of 7.06. The *C* terminus of this protein possesses the classic characteristics of the A, B, C and D motifs of the CytP450 family: phenylalanine (F) and glycine–glycine (GG) residues are highly conserved in structural domain A; structural domain B has a highly conserved leucine (L) and proline (P); the cysteine in structural domain D shows the highly conserved characteristics of a hemoglobin-binding site (Figure 2).

BLAST analysis of the amino acid sequence of the *CsHPL*-encoding region was conducted to identify homologous plant HPL amino acid sequences. The deduced nucleotide sequence of the *CsHPL* gene showed 99% homology with the *CsHPL* gene (AF229811.1) cloned from the hypocotyls of cucumber (*Cucumis sativus* L. cv Suyo) by Mastkui *et al.* [8] and 95% homology with the *9-HPL* gene (AF081955.1) cloned from melon fruits (*Cucumis melo* L. cv. Caravelle) by Tijet *et al.* [13]; further, these sequences showed 99% and 94% amino acid sequence homology, respectively. The amino acid sequence of the *CsHPL* gene showed 51% homology with that of the AOS protein in melon (*Cucumis melo* L.; AAM66138.1). In addition, the amino acid sequences of *HPL* and *AOS* gene are highly homologous to hemoglobin-binding sites, both of which harbor aspartate-lysine-glutamine (NKQ) upstream of cysteine. Therefore, the cucumber *HPL* gene amino acid sequence is highly homologous to the *AOS* gene amino acid sequences of many plants, as exemplified by the 68% homology with the *AOS* gene amino acid sequence of potato. The downstream sequence of cysteine (C) is also conserved (Figure 2).

**Figure 3:**
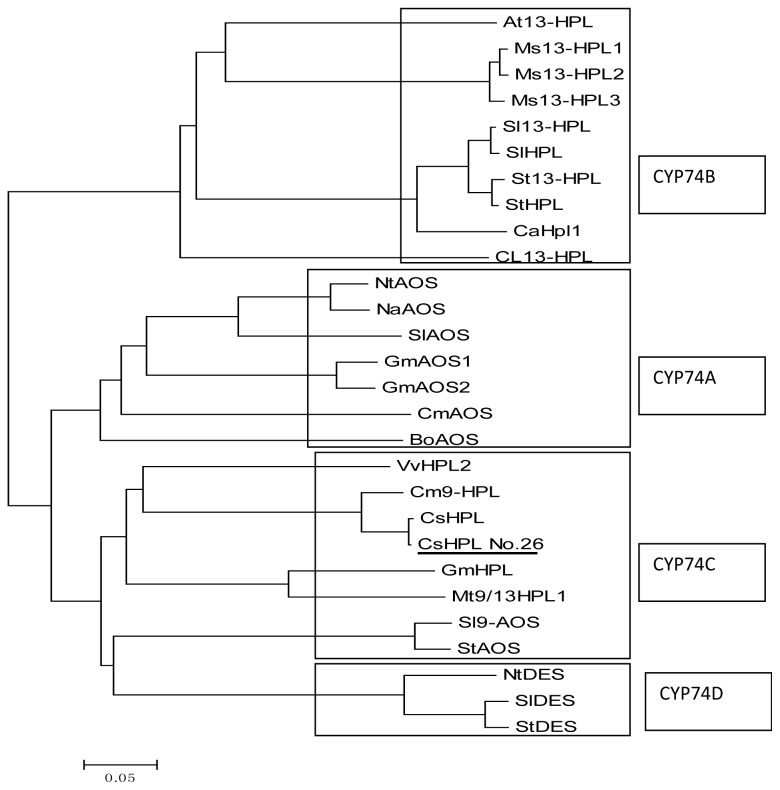
Phylogenetic tree based on the deduced amino acid sequences of plant *HPL* genes. The nucleic acid sequences used are listed below with GenBank accession numbers: VvHPL2: *Vitis vinifera* ADP88811.1; GmHPL: *Glycine max* AGH32771.1; Mt9/13HPL: *Medicago truncatula* CAC86899.1; NtAOS: *Nicotiana tabacum* BAM76723.1; BoAOS: *Brassica oleracea* AGB34186.1; At13-HPL: *Arabidopsis thaliana* AAC69871.1; Ms13-HPL1 CAB54847.1; Ms13-HPL2: *Medicago sativa* CAB54849.1; Ms13-HPL3: *Medicago sativa* CAB54848.1; Sl13-HPL: *Solanum lycopersicum* AAF67142.1; St13-HPL: *Solanum tuberosum* CAC44040.1; GmAOS1: *Glycine max* ABB91776.1; GmAOS2: *Glycine max* ABB91777.1; NaAOS: *Nicotiana attenuata* CAC82911.1; SlAOS: *Solanum lycopersicum* AAF67141.1; NtDES: *Nicotiana tabacum* AAL40900.1; SlDES: *Solanum lycopersicum* AAG42261.1; StDES: *Solanum tuberosum* CAC28152.1; Sl9-AOS: *Solanum lycopersicum* AAN76867.1; StAOS: *Solanum tuberosum* ABA54984.1; Cm9-HPL: *Cucumis melo* AAK54282.1; CsHPL: *Cucumis sativus* AAF64041.1; CsHPL No. 26: CsHPL from No. 26; CmAOS: *Cucumis melo* AAM66138.1; SlHPL: *Solanum lycopersicum* CAB43022.1; CaHPL1: *Capsicum annuum* AAK27266.1; StHPL: *Solanum tuberosum* ACT64589.1; Cl13-HPL: *Citrullus lanatus* AAU12570.1.

A phylogenetic tree was constructed to determine the evolutionary placement of CsHPL protein sequences in relation to the other plant CYP74 sequences (Figure 3) using Mega5 software. HPL proteins of 24 plant species were divided into 4 categories: CYP74A, CYP74B, CYP74C and CYP74D. Within the tree, CsHPL from No. 26 is grouped closely with CYP74C subfamily whose members are classified as 9/13-HPLs. CsHPL (*Cucumis sativus* L. cv. Suyo), Cm9-HPL (*Cucumis melo* L. cv. Caravelle), VvHPL2 (*Vitis vinifer*a L. Cabernet Sauvignon), and GmHPL (soybean) are closely related in this phylogenetic tree, and CsHPL is also closely related to StAOS and SlAOS, which show AOS activity.

### 2.2. Expression of *CsHPL* and C6/C9 Aldehyde Accumulation in Developing Cucumber Fruit

#### 2.2.1. The Expression of *CsHPL* during Fruit Development

**Figure 4:**
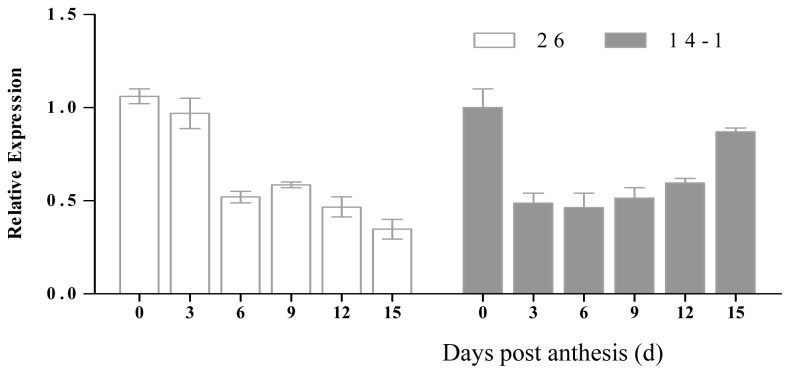
Expression levels of the *CsHPL* gene in No. 26 (an inbred line of Northern China) and *CsHPL* gene in No. 14-1 (an inbred line of Southern China) during fruit development post-anthesis. Expression levels of fruit *CsHPL* genes were analyzed by using quantitative real-time PCR during cucumber fruit development after anthesis. *Y* axis represents relative expression values. Data represent the means ± SD of three biological replicates (fruits from three plants were taken as a biological replicate). The expression of the genes was normalized with reference to the expression of the *actin* gene.

The expression mode of *CsHPL* was different in different inbred lines during fruit development. *CsHPL* expression in inbred line No. 26 was the highest at 0 dpa, and then declined to the lowest level at 15 dpa. Its expression was the highest at the early stage of fruit development and in female flowers. The expression of *CsHPL* in inbred line No. 14-1 was the highest at 0 dpa, and then declined to the lowest at 6 dpa and increased gradually, following a parabolic pattern. The difference in the expression pattern in the two inbred lines could be attributed to the difference in genotypes (Figure 4).

#### 2.2.2. HPL Enzyme Activity during Fruit Development

**Figure 5:**
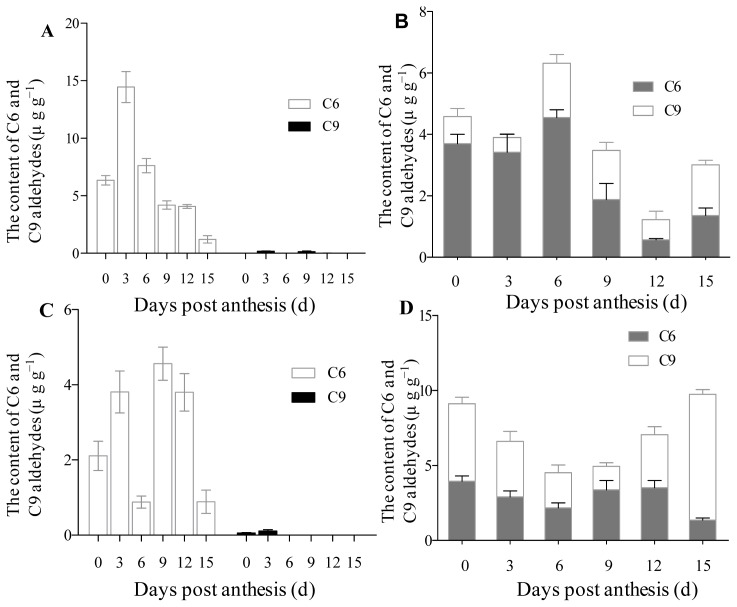
C6 and C9 aldehyde contents produced by crude enzyme extracts from cucumber fruit at different development stages using HPOD as substrate. (**A**) C6 and C9 aldehyde contents produced using 13-HPOD as a substrate during cucumber fruit development in No. 26; (**B**) C6 and C9 aldehyde contents produced using 13-HPOD and 9-HPOD mixture as a substrate during cucumber fruit development in No. 26; (**C**) C6 and C9 aldehyde contents produced using 13-HPOD as substrate during cucumber fruit development in No. 14-1; and (**D**) C6 and C9 aldehyde contents produced using 13-HPOD and 9-HPOD mixture as substrate during cucumber fruit development in No. 14-1. *Y* axis represents C6 and C9 aldehydes contents. Data represent the means ± SD of three biological replicates (three fruits were taken as a biological replicate).

The substrates 13-HPOD and 13-HPOD + 9-HPOD mixtures were put in a closed reaction system respectively and then cleaved using the crude enzyme extracted from cucumber fruits of different development stages in order to identify the HPL enzyme activity of the fruit at different development stages. Then the contents of C6 and C9 aldehydes were analyzed by gas chromatography-mass spectrometry (GC-MS). Because 13-HPL catalyses 13-HPOD or 13-HPOT to produce hexanal and hexenals respectively, while 9-HPL uses 9-hydroperoxides as substrates to form C9 aldehydes, the contents of C6 and C9 aldehydes produced can reflect the HPL enzyme activity. The 13-HPL activity was found to be higher at 0–6 dpa in the inbred line No. 26, and then decreased to the lowest level at 15 dpa. The total enzyme activity was the highest at 6 dpa. C6 aldehydes were mainly produced when the substrate was cleaved by the crude enzyme of 0–6 dpa, whereas the proportion of C9 aldehydes increased when the substrate was cleaved by the crude enzyme of 9–15 dpa. Hence, the inbred line No. 26 mainly showed 13-HPL activity at the early development stage (0–6 day), and predominant 9-HPL activity at the later development stage (9–12 day). On the other hand, 13-HPL activity was the highest at 6 dpa in the inbred line No. 14-1; the total enzyme activity was higher at 0 and 15 dpa and was the lowest at 6 dpa. The proportion of C9 aldehydes was higher in this inbred line when the substrate was cleaved by the crude enzyme of 0 and 15 day fruit post-anthesis (Figure 5A–D).

#### 2.2.3. Changes of C6 and C9 Aldehyde Content during Cucumber Fruit Development

**Figure 6:**
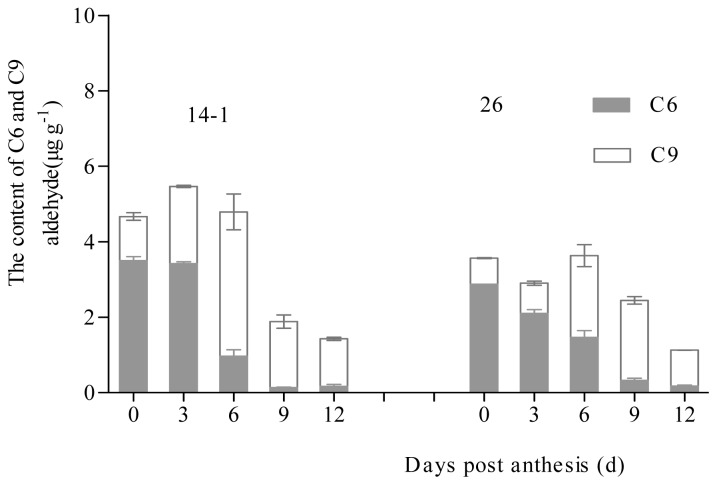
Changes in C6 and C9 content of cucumber fruits during fruit development. Content of C6 aldehydes and corresponding alcohols in developing cucumber fruits. Data represent the means ± SD of three biological replicates (fruits from three plants were taken as a biological replicate).

The C6 and C9 aldehydes and relevant alcohols were the main components of cucumber fruit, in which certain C6 aldehydes and C6 alcohols, such as hexanal, (*E*)-2-hexene, hexanol, (*E*)-2-hexene-1-ol, (*E*)-3-hexene-1-ol, had “grass” and “green note” odors. On the other hand, C9 aldehyde and relative C9 alcohols, such as (*E*,*Z*)-2,6-nonadienal, (*E*)-6-nonene aldehyde, (*Z*)-6-nonene aldehyde, nonanal, (*Z*)-6-nonen-1-ol, (*E*,*Z*)-2,6-nonadien-1-ol, and (*E*,*Z*)-3,6-nonadien-1-ol had “cucumber like” and “flower like” odors. Therefore, these two main compounds have an important effect on the flavor of cucumber. The contents of C6 aldehydes and C6 alcohols were higher at the early stage of development and declined significantly at 6 dpa; only a small proportion of aldehyde and relative alcohols were present at 9–12 dpa. Unlike C6 aldehyde and C6 alcohol contents, the contents of C9 aldehyde and C9 alcohols were lower at the early stage of development and increased significantly at 6 dpa; a large proportion of this aldehyde and relative alcohols was found at 9–12 dpa (Figure 6).

#### 2.2.4. Coordination among HPL Enzyme Activity, *CsHPL* Gene Expression, and Aldehyde Content during Cucumber Fruit Development

**Figure 7:**
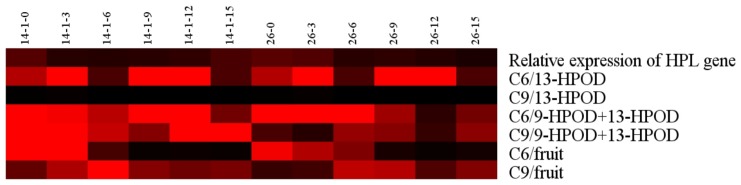
The heat map about the *CsHPL* gene expression, enzyme activity of fruit identified by the catalytic action of crude enzyme extracts incubating with *n*-HPODs and aldehyde production during fruit development in both lines. Relative expression levels of the *CsHPL* gene, C6 or C9 content at each sample point are shown by the colour scale. The lowest content levels are in black, and the highest in red. 14-1-0 to14-1-15 refer to the six developmental stages of fruits in No. 14-1 lines, and 26-0 to 26-15 refer to the six developmental stages of fruits in No. 14-1 lines. C6/13-HPOD (C9/13-HPOD): C6 (C9) aldehyde contents produced using 13-HPOD as a substrate during cucumber fruit development in two lines; C6/9-HPOD + 13-HPOD (C9/9-HPOD + 13-HPOD): C6 (C9) aldehyde contents produced using 13-HPOD and 9-HPOD mixture as a substrate during cucumber fruit development in two lines. C6/fruit (C9/fruit): C6 (C9) aldehyde contents produced during fruit development.

The relationship between HPL enzyme activity and *CsHPL* gene expression in the two inbred lines showed that *CsHPL* gene expression peaked at 0 and 3 dpa in inbred line No. 26 and declined to the lowest level at 15 dpa. However, the total HPL activity peaked at 6 and 12–15 dpa; the 13-HPL activity peaked at 6 dpa and declined to lower levels at 12–15 dpa. The expression peak of *CsHPL* gene preceded that of HPL during the fruit development. *CsHPL* gene expression in inbred line No. 14-1 showed a parabolic pattern during fruit development, and the expression pattern of the total enzyme activity was synchronized to the gene expression mode. The content of C6 aldehyde and C6 alcohols was higher during the early stages of fruit development (0–3 dpa), decreased significantly at 6 dpa, and only accounted for a small proportion of fruit aldehydes (mainly C9 aldehydes) at 9–12 dpa (Figure 7).

#### 2.2.5. The Expression of *CsHPL* in Different Tissues and Organs

**Figure 8:**
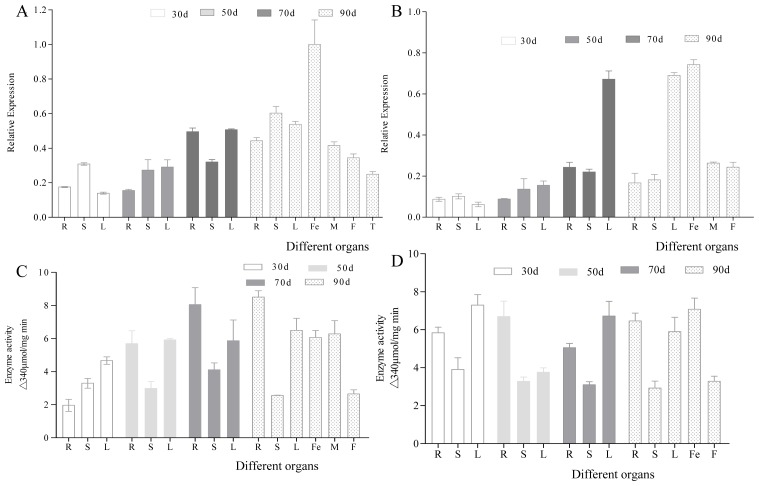
*CsHPL* gene expression and HPL enzyme activity in the different organs of cucumber. (**A**) The relative expression of *CsHPL* gene in No. 26; (**B**) The relative expression of *CsHPL* gene in No. 14-1; (**C**) The 13-HPL enzyme activity in No. 26; and (**D**) The 13-HPL enzyme activity in No. 14-1. The bars represent gene relative expression or enzyme activity in different organs of cucumber. The error bars are the standard deviations from three biological repeats. R, Root; S, Stem; L, Leaf; Fe, Female flower; M, Male flower; F, Fruit; T, Tendril.

The expression of *CsHPL* was highest in female flowers and lowest in the roots at 30 days (Figure 8A,B); this finding is consistent with that of Matsui *et al.* [21]. In addition, *CsHPL* expression was upregulated with the growth and development of cucumber plants. High levels of *CsHPL* gene expression were mainly noted in the leaves, stems, and reproductive organs at 90 days.

#### 2.2.6. 13-HPL Enzyme Activity in Different Tissues and Organs

The 13-HPL enzyme activity was analyzed using UV spectrophotometry (Figure 8C,D) and was detected in all tissues and organs of both inbred lines: it was the highest in 50-day-old female flowers, male flowers, roots, and leaves.

#### 2.2.7. *CsHPL* Expression during Gray Mold Infection

**Figure 9:**
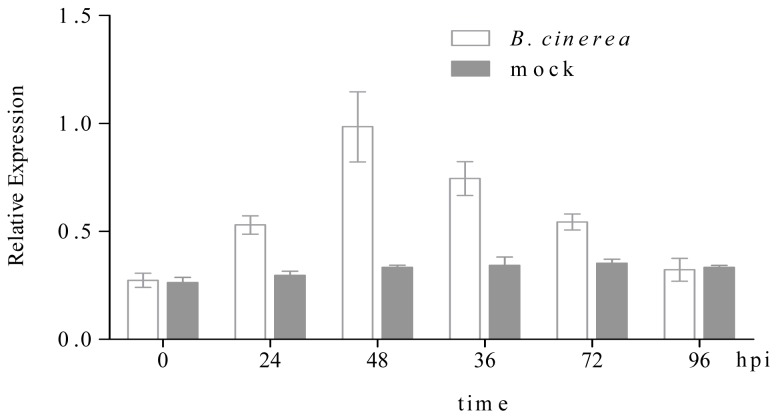
Expression of the *CsHPL* gene in No. 26 cucumber plants infected with *Botrytis cinerea.* Expression of the *CsHPL* gene in No. 26 cucumber plants infected with *B. cinerea*. The leaves of control plants were sprayed with mock solution (grey bars) or *B. cinerea* spores (white bars) and were harvested at the indicated time points. *CsHPL* expression levels were quantified by real-time polymerase chain reaction (PCR) and expressed as normalized expression to the cucumber reference gene *actin* expression level. Data are the mean of three independent experiments. hpi: hour post inoculation. *B. cinerea: Botrytis cinerea*; mock, mock soluthion.

Cucumber seedlings at the two-leaf stage were inoculated with gray mold and sampled at 0, 24, 48, 36, 72 and 96 hpi (hour post inoculation) to investigate the response of the *CsHPL* gene to gray mold infection. *CsHPL* expression increased 24 h after gray mold inoculation and then gradually decreased, indicating the elicitation of its response within 24 h after gray mold infection (Figure 9).

### 2.3. Determination of Prokaryotic Expression and Recombinase

**Figure 10:**
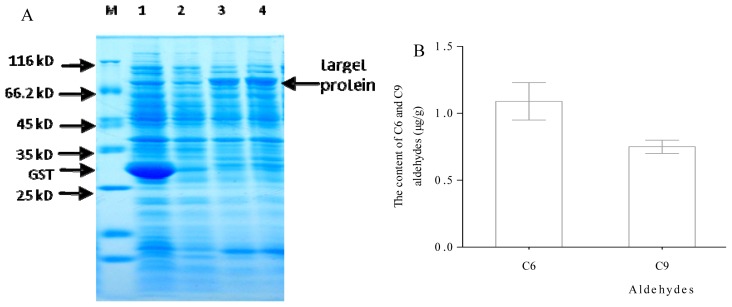
Expression of recombinant CsHPL protein and analysis of recombinant enzyme using GC-MS. (**A**) Expression of recombinant CsHPL protein. **M**: Protein Marker; **1**: blank vector induced with 0.2 mM IPTG; **2**: recombinant vector induced with 0.1 mM IPTG; **3**: recombinant vector induced with 0.2 mM IPTG; **4**: recombinant vector induced with 0.3 mM IPTG; and (**B**) Analysis of the recombinant enzyme using GC-MS.

IPTG was used as an inducer for induced expression analysis. According to the size of *CsHPL* ORF and tag, the recombinant proteins expressed in *E. coli* were predicted to be approximately 80 kD. When the cells were incubated with 0.3 mmol·L^−1^ IPTG for 5 h at 37 °C, the 80 kD protein was detected (Figure 10), which is consistent with the expected results.

Recombinase activity of CsHPL was measured by assessing its ability to cleave the substrate 13-HPOD + 9-HPOD mixture using GC-MS. The average contents of C6 and C9 aldehydes were 0.95 and 0.7, respectively, indicating that CsHPL recombinase had both 13-HPL and 9-HPL activities. The sequence analysis also showed that this gene belongs to the CYP74C subfamily, and the GC-MS analysis results confirmed that the *CsHPL* gene is the *9/13HPL* gene of cucumber. The contents of C6 and C9 aldehydes detected by GC-MS indicated that CsHPL recombinase has a 13-HPL enzyme preference.

### 2.4. Discussion

#### 2.4.1. Sequence Characteristics of Cucumber *HPL* Gene

Recently, data from cucumber genome sequencing indicated that there are 3 *HPL* genes in its genome [31]. The complete sequence of one of the *HPL* genes, *i.e.*, *9/13-HPL* (Accession No. AF229811.1), which is specifically expressed in vegetative organs such as the roots, hypocotyls, and leaves, is available. Homology cloning of this gene was conducted using RNA extracted from the cotyledons and fruits of plants from the No. 26 and 14-1 inbred lines respectively. Sequence analysis indicated *CsHPL* and *CsHPL* display 99% homology to the cucumber *9/13-HPL* gene (AF229811.1), 95% homology to the melon *9-HPL* gene (Accession No. AF081955.1), as well as 4 conserved regions of the classic plant *HPL* gene, indicating features common to the cytochrome CYP74D family. According to the classification of Noordermeer *et al.* [9] and the sequence characteristics, the cytochrome CYP74 family can be divided into 4 types: CYP74A(AOS proteins); CYP74B (13-HPLs proteins); CYP74C (9/13-HPLs proteins); and CYP74D (divinyl ether synthase proteins). On the basis of substrate specificity, HPL proteins can be divided into 3 types: 13-HPL, 9-HPL and 9/13-HPL. Previous studies have shown that 9/13-HP mainly exists in cucumber fruits and seedlings, as well as alfalfa seedlings [8,32]. Sequence analysis indicated that the *CsHPL* gene encodes a 9/13-HPL protein in Northern Chinese cucumber germplasms.

#### 2.4.2. Expression Pattern and Characteristics of the *HPL* Gene during Fruit Development

Expression of *HPL* genes in plants has been noted throughout the growth and various development stages, including seed germination, tuber formation, ripening, and senescence [5,17,23]. *HPLs* have also been reported to participate actively in defense responses to biotic and abiotic stresses and the conversion of HPOD or HPOT to aldehydes, contributing to the synthesis of C6 and C9 aldehydes [33,34]. In this study, we found that expression of *CsHPL* and *CsHPL* was regulated by fruit development stages and genetics, which have also been confirmed in other cucumber cultivars [21], *Arabidopsis* [4], tomato [16], lemon [35], and apricot [17]. For example, it was reported that the expression of *9/13-HPL* was regulated in cucumber by development, and its expression level was higher in the hypocotyls, female flowers, and fruits, and lower in mature leaves [21]. In addition, its expression pattern varied among different germplasms. The HPL level was found to peak at 23 and 28 weeks after flowering in different apricot germplasms “Arbequina” and “Picual”, respectively [17].

#### 2.4.3. Relationship of *HPL* Gene Expression, Enzyme Activity, and Aldehyde Production during Fruit Development

Real-time polymerase chain reaction (PCR) analysis showed that *CsHPL* gene expression was higher at the early stages of fruit development, and that total HPL and 13-HPL activities gradually increased from 0 dpa up to the highest level at 6 dpa. This result is consistent with that of Liu *et al.* [33] who showed that HPL activity increased from the early stage of development up to the completion of cucumber fruit development. Matsui *et al.* [21] showed that 13-HPL activity decreased with fruit development and decreased to the lowest level when the fruits were ripe. The 9-HPL activity followed a parabolic pattern and was consistent with that of the total enzyme activity of the No. 14-1 inbred line.

Our results indicate that the HPL activity was not entirely synchronized with the expression pattern of the *CsHPL* gene. A patterning was observed between the total content of volatile C6 and C9 aldehydes and alcohols and HPL enzyme activity. In inbred line No. 26, the total content changes of C6 and C9 aldehydes and alcohols were consistent with those of total HPL activity, both of which peaked at 6 dpa as was noted for *CsHPL* gene expression. The total content of C6 aldehydes and corresponding alcohols was consistent with that of the total enzyme activity during the early stage of fruit development (0–6 day) in inbred line No. 14-1, and C9 aldehydes and relative alcohols were the main volatiles at 6 dpa. Liu *et al.* [5] reported that HPL activity was higher during the early stages of fruit development, peaked at 6 dpa, and then decreased, which is consistent with the results obtained in this study. In the LOX-HPL metabolic pathway, the content of C6 aldehydes was higher during the early stages of cucumber fruit development, and then decreased as fruit development progressed; this was consistent with the changes in HPL activity. The decomposition of unsaturated fatty acids requires both LOX and HPL to produce C6 aldehydes; therefore, the content of aldehydes is closely related not only to HPL activity but also to LOX activity.

## 3. Experimental Section

The materials used in this study were inbred line No. 26 and inbred line No. 14-1. No. 26 was selected from high generation inbred lines of a Northern China type cultivar “Jinyan No.1”, and inbred line No. 14-1 was selected from high generation inbred lines of a Southern China type cultivar “Deltastar”. No. 26 has a relatively high 2,6-nonadienal/(*E*)-2-nonenal ratio (2.778), resulting in an intense “cucumber-like” flavor. No. 14-1 has a relatively low 2,6-nonadienal/(*E*)-2-nonenal ratio (1.414). Both No. 26 and 14-1 are the backbone parental ones used by our research group for breeding. The plants were seeded on 20 February 2011 and cultured in substrate using 50-hole cell trays. The seedlings which had 3 or 4 true leaves were transplanted into the plastic tunnels in the garden station of the College of Horticulture, Northwest A&F University (Xi’an, China) in 15 March 2011. For the sampling of the different organs, 30-, 50-, 70- and 90-day-old plants which were selected randomly, and the root, stem, leaf, male and female flower were sampled respectively after the roots were washed carefully and dried using blotting paper. For the sampling of the different fruit development, the opening female flowers that were at approximately the same node (14th node) were selected and marked. Fruits of 0, 3, 6, 9, 12 and 15 dpa were sampled and three biological replicates of three plants were taken, and the fruits were quick-frozen in liquid nitrogen and maintained at −80 °C until analysis.

### 3.1. Total RNA Extraction, Purification, and cDNA Synthesis and Cloning

The RNAs of various cucumber tissues and organs were extracted using an RNA rapid extraction kit (Beijing Tiandz Inc., Beijing, China). The concentrations and purities of the RNA were determined using NanoDrop (NanoDrop Technologies, Wilmington, DE, USA), and the integrity was tested using agarose gel electrophoresis after the extracted RNA had been treated with *DNase*I (MBI Fermentas, St. Leon-Rot, Germany). Reverse transcription was performed using approximately 1 μg of purified RNA as template with oligo(dT)_18_ and a Revert Aid First Strand cDNA Synthesis Kit (MBI Fermentas), as per the manufacturer’s instruction.

The plasmid extraction kit was purchased from OMGA (Omega Bio-Tek Inc., GA, USA); the gel extraction kit, from TaKaRa (TaKaRa, Kyoto, Japan); and the enzymes and reverse transcription kit, from Fermantas. The spectrophotometry was obtained from Thermo Scientific Evolution 300 (Thermo Fisher Scientific Inc., Waltham, MA, USA). The gas chromatography-mass spectrometry (GC-MS) instrument was purchased from TRACE DSQ (Thermo-Finnigan, San Jose, CA, USA).

For cucumber *HPL* gene cloning, the cucumber *HPL* gene sequence registered in NCBI (Accession No. AF229811) was used to design the primers *HPL-*F and *HPL-*R using the software Primer5.0 (Premier Biosoft International, Palo Alto, CA, USA). The primer sequences are as follows: *HPL-*F, 5′-ATGGCTTCTTCCTCCCCTGAA-3′; *HPL-*R, 5′-TTAGCTTCTAAACCGAAGCGG-3′. The PCR amplification conditions were as follows: 94 °C for 1 min, followed by 30 cycles of 94 °C for 30 s; 51 °C for 30 s, and 72 °C for 1 min, and a final extension at 72 °C for 3 min.

For the separation of PCR products, 1.2% agar gel electrophoresis was conducted, and target bands were excised, recovered, and purified. The products were then attached to T-easy cloning vectors for transformation of DH5α competent cells. Three correct clones of recombinant plasmids were selected through PCR screening of the bacterial suspension and sent to Shanghai Aoke Biological Company, Ltd. (Shanghai, China) for sequencing.

### 3.2. Sequence Analysis

Sequence analysis and prediction of protein-conserved regions of cucumber *CsHPL* were conducted using BLAST [36]. BLASTP was conducted using DNAman software (Lynnon BioSoft, Vaudreuil, Canada), and cluster analysis was performed using Mega5 [37].

The Protparam software [38] was used to analyze the physiochemical characteristics of the *CsHPL* protein product, including its relative molecular weight, isoelectric point (pI), and stability. Network protein sequence analysis [39] was applied to predict the secondary structure of cucumber *CsHPL*. The online program Psort I [40] was used to analyze the subcellular localization of the protein encoded by *CsHPL*.

### 3.3. Prokaryotic Expression of *CsHPL* and Enzymatic Activity Analysis of Recombinase

The cucumber *CsHPL* gene was used as a template to design the upstream primer *Y-HPL-*F 5′-CCGCTCGAGATGGCTTCTTCCTCCCCTGA-3′ and downstream primer *Y-HPL-*F 5′-ATAAGAATGCGGCCGCTTAGCTTCTAAACCGAAGCGG-3′, in which the underlined sequences were the endonuclease sites for *Xho*I and *Not*I. The primers were extended by *Xho*I and *Not*I restriction sites for ligation into the bacterial expression vector *pGEX-4T-1* using T4 DNA ligase. This vector provides a start codon, and a GST tag is added to the *N* terminus of the HPL protein. The resulting 1.4 kb PCR product was digested using the restriction enzymes and ligated into *Xho*I- and *Not*I-digested and dephosphorylated *pGEX-4T-1. E. coli* strain DH5α was transformed with this plasmid and selected on LB plates. Recombinant plasmids were checked with *Xba*I and *Pst*I and sequenced.

A single positive colony of freshly transformed bacteria was inoculated into LB liquid medium containing 100 mg·L^−1^ ampicillin and grown at 37 °C until the A_600_ was 0.5. Next, 200 mL of LB ampicillin medium was inoculated with the entire pre-culture and grown at 37 °C. When the culture reached an A_600_ of 0.6, 0.6 mmol·L^−1^ IPTG was added to induce gene expression. The culture was subsequently incubated at 25 °C for 3 h, and the samples were collected at 0 and 3 h. Approximately 1.5 mL aliquots of the culture suspension were harvested by centrifugation at 2500 × *g* for 10 min at 4 °C. Cell pellet aliquots corresponding to 50 mL cultures were washed with water, frozen with liquid nitrogen, and maintained at −80 °C.

Crude recombinase was prepared according to the separation method described by Makstui *et al.* [8,20], with modifications. Cells were collected at 4 °C, washed with 50 mM PBS (phosphate buffered saline, pH 7.0) buffer three times, centrifuged and suspended in Buffer A (50 mM Tris-HCl, 2 mg/mL lysozyme, pH 8.0), shaken at 4 °C for 20 min, and then centrifuged to obtain protoplasts. The protoplasts were then treated with Buffer B [50 mM Tris-HCl, 500 mM NaCl, 0.5% Triton X-100, 0.5 mM PMSF (phenylmethylsulfonyl fluoride, pH 8.0), lysed using ultrasonification (380 W, 1 s with 2 s intervals) for 3 min, and then centrifuged at 4 °C for 20 min. The cell fragments were disposed, and the supernatant was collected as the crude enzyme solution of HPL recombinase.

13-HPOD and 13-HPOD + 9-HPOD mixtures were used as the reaction substrates. The reaction system included 50 μL of the crude recombinase solution, 84 μL of the substrate, 10 μL of 10 mM NADH (nicotinamide adenine dinucleotide), and 10 μL of 5 U·μL^−1^ ADH; this was diluted to 1 mL using 0.5 mmol·L^−1^ PBS buffer (pH 6.0). The reaction product was directly added to the extraction bottle, and the C6 and C9 aldehyde contents were measured using GC-MS, which indirectly reflected the total HPL activity of cucumber fruit 13-HPL. Preparation of 13-HPOD was performed according to Gargour’s method [41]. The LOX enzyme solution was purchased from Sigma (Sigma-Aldrich, St. Louis, MO, USA) and maintained at 4 °C, and borate buffer (pH 8.9) was added when preparing the substrates. The LOX enzyme solution used in the 13-HPOD + 9-HPOD mixture was extracted from potatoes following the method described by Vick [42].

### 3.4. HPL Activity Analysis during Fruit Development

Fruits from 0, 3, 6, 9, 12 and 15 dpa were sampled, transported to the laboratory on ice, and the following treatments were immediately performed. The top, middle, and bottom parts of the cucumber fruit were sampled, weighed to approximately 2–5 g, and mixed. The samples were ground in a mortar with twice the volume of extr*actin*g solution [0.1 mol·L^−1^ PBS (pH 6.5), 0.5% PVP-K 30, and 0.5% Triton X-100] and 2 μL of 0.5 mM PSMF protein inhibitor at 4 °C. After thorough grinding, the mixture was centrifuged at 12,000 rpm for 30 min at 4 °C, and the supernatant containing the crude recombinase extract was collected.

### 3.5. Expression Analysis of *CsHPL* in Various Cucumber Tissues and Fruits Collected on Different Days after Flowering

Real-time PCR was used to analyze the expression of the *CsHPL* gene at 0, 3, 6, 9, 12 and 15 dpa. Diluted cDNA was used as the template, and the cucumber *actin* gene was used as the reference gene. The real-time quantitative PCR system was prepared following the instructions of the real-time PCR kit manufactured by TaKaRa. BioRad IQ5 (Bio-Rad, Hercules, CA, USA) was used as the PCR amplifier. The primer sequences of *CsHPL* genes were as follows: *CsHPL*-RT F, 5′-CTCCTTTCTCGCTTCTCACC-3′; and *CsHPL*-RT R, 5′-CTCAAACGACACGGCATCACT-3′. The primer sequences of the *actin* gene were *Cu-actin* F 5′-TCGTGCTGGATTCTGGTG-3′ and *Cu-actin* R 5′-GGCAGTGGTGGTGAACAT-3′. The reaction procedure was as follows: 95 °C for 1 min, followed by 40 cycles of 95 °C for 15 s, 57 °C for 30 s, and 72 °C for 20 s. A solubility curve was drawn within the range of 55–95 °C, with 0.5 °C increase at 15 s intervals. Fluorescence signals were recorded at 72 °C. The procedure was performed three times for each sample, and the average was obtained.

### 3.6. C6 and C9 Aromatic Aldehyde Content Testing during Cucumber Fruit Development

Measurement of volatiles was performed according to Zhang *et al.* [40], with modifications. A total of 10 g of flesh tissue of cucumber was chopped quickly, ground in liquid nitrogen, transferred to a 10 mL vial, and 1 g sodium chloride of analysis grade was added. Ten microliters of octanal solution (1 μL/mL) was used as an internal standard. The vials were sealed and then stirred for 10 s using a vortex (Kylin-Bell Lab Instruments Co., Ltd., Haimen, China).

For manual solid-phase microextraction (SPME) analysis, the extraction fiber was aged for 2 h at 250 °C in a GC-MS injector (Supelco, Deisenhofen, Germany). The aged fiber was pushed into the head-space part of vials to extract volatiles in a 40 °C water bath for 30 min, and then the volatiles were desorbed for 3 min at 230 °C into the splitless injection port of the GC-MS. A HP-INNOWax column (0.25 mm, 60 m, 0.25 μm; Agilent Technologies, Waldbronn, Germany) was used for volatile analysis. Chromatography conditions were as follows: initial oven temperature, 40 °C held for 2.5 min, increased by 6 °C·min^−1^ to 230 °C, and then held for 7 min. Nitrogen was used as a carrier gas at 1.0 mL·min^−1^.

Volatiles were identified by comparing the retention times with those of authentic standards (ion source, 250 °C; electron energy, 70 eV; multiplier voltage, 1.4 kV; and scan range, 35–400 mass units). Volatiles were identified by comparing their electron ionization (EI) mass spectra with those of published data and data from authentic standards.

Quantitative determination of compounds was carried out using the peak of the internal standard as a reference value and calculated on the basis of the standard curves of authentic compounds [43].

The quantitative determination of compounds was as follows.

Content of volatiles compound (μg·g^−1^) = peak area of compounds/(peak area of internal standard × mass of the sample (g) × mass concentration of internal standard (μg·μL^−1^) × volume of internal standard (μL).

The identity of odor impact compounds: the compounds that had volatile values greater than 1 were considered as the odor impact compounds [44].

The heat map analysis was done on a MultiExperiment Viewer program available at [45]. The relative expression levels of the *CsHPL* gene, C6 or C9 content at each sample point are shown by the colour scale.

### 3.7. Gene Expression Analysis of Gray Mold Infection on Cucumber Seedling Leaves

The gray mold strains were provided by Professor Qing Ma of the College of Plant Protection, Northwestern A&F University. Gray mold bacteria (*Botrytis cinerea*) were cultivated in PDA. When the Petri dish was completely filled with hyphae, the cultivated conidia were resuspended in sterilized distilled water containing Tween-20. With the aid of a microscope, the concentration of the conidial suspension was adjusted to 1.0 × 10^6^ conidia mL^−1^ before inoculation. Cucumber seedlings were planted in nutrition containers, and the culture medium was sterilized using moist heat. Cucumbers were cultivated in a phytotron at a temperature of 25 °C/20 °C and illumination of 7000 lx. Gray mold inoculation was performed when the first true leaves appeared on a seedling. Using a pipette, approximately 2 μL of the conidial suspension was inoculated onto the first leaves at the bottom of a cucumber seedling, while approximately 2 μL of mock solution (the distilled water containing Tween-20) was inoculated onto the first leaves of control seedling, and a plastic bag was placed over the seedling to retain moisture. Humidity was maintained above 90% for 24 h before inoculation and to 100% for 48 h after inoculation; the temperature was maintained at 25 °C/20 °C. In this study, 40 inoculated seedlings and 40 controls were used. The whole plant was collected as the sample at 0, 24, 36, 48, 72 and 96 h after inoculation. Two samples were collected for each test material: one sample was quickly frozen in liquid nitrogen and stored at −80 °C in a fridge until gene expression profiling using real time-PCR, and the other sample was used for the analysis of enzyme activity.

## 4. Conclusions

In conclusion, the *HPL* gene from cucumber fruit was isolated and characterized. Sequence analysis of the *CsHPL* gene showed that it encoded a 9/13-HPL protein. The identity of the *HPL* gene was confirmed by functional expression in bacteria. The recombinant cucumber HPL preferentially exhibits 13-HPL enzyme activity. *CsHPL* expression is spatially and temporally regulated during the development and ripening of cucumber fruit. All these data indicate the involvement of *CsHPL* in the biosynthesis of aromatic aldehyde compounds.
